# Ophthalmic Shingles in Children: A Rare Form and a Danger to Be Aware of

**DOI:** 10.7759/cureus.61408

**Published:** 2024-05-31

**Authors:** Hassnae Tkak, Aziza Elouali, Ayad Ghanam, Abdeladim Babakhouya, Maria Rkain

**Affiliations:** 1 Department of Pediatrics, Mohammed VI University Hospital, Faculty of Medicine and Pharmacy, Mohammed I University, Oujda, MAR

**Keywords:** immunity, complication, herpes virus, ophthalmic shingles, child

## Abstract

Shingles in children is rare, especially the ophthalmic form. The occurrence of shingles in children is exceptional but often benign, requiring only symptomatic treatment. Antivirals are exceptionally used for complicated forms, which are mainly seen in immunocompromised individuals or in ophthalmic locations that can lead to severe ocular complications. Various studies agree on the benign nature of this condition in immunocompetent children and an excellent prognosis. We report a case of ophthalmic shingles in an immunocompetent child aged two years and seven months. The purpose of this clinical case was to emphasize the importance of early antiviral treatment to limit corneal involvement and preserve visual function.

## Introduction

Shingles is a viral disease caused by the varicella-zoster virus (VZV), a DNA virus belonging to the herpesviridae family, which is strictly transmitted between humans. This virus causes chickenpox during the primary infection or shingles when the VZV reactivates after remaining latent in the Gasserian ganglion following an initial chickenpox infection. It has a particular affinity for the skin, nervous system, and lungs [1]. Ophthalmic shingles is an uncommon clinical manifestation in children and mainly affects older individuals due to a weakened immune system associated with aging. This explains why shingles is more frequent in the elderly. Ophthalmic shingles can lead to severe ocular complications that require appropriate and early management [2].

## Case presentation

The patient is a two-year-and-seven-month-old girl with a history of a primary varicella infection two weeks ago, chickenpox in her sister one month ago, and no skin rash observed in the mother in the days before or after delivery. Otherwise, she had good growth and weight development and no history of recurrent infections.

**Figure 1:**
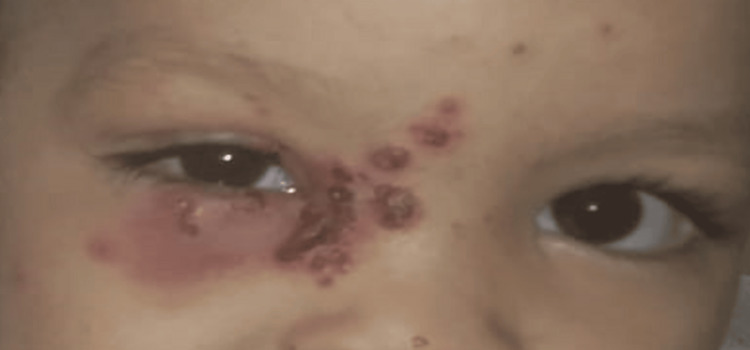
Multiple grouped vesicles, resting on erythematous skin, affecting the right side of the nose, the right half of the forehead with edema of the upper and lower right eyelids with difficulty in opening the eye

For two days prior to admission, she had been presenting with skin lesions and eyelid edema of the right eye, occurring without fever. The clinical examination at admission found the child in good general condition, afebrile, with multiple vesicles grouped in clusters and honey-colored crusts on erythematous skin affecting the inner corner of the eye, the right side of the nose, and eyelid edema limiting the eye opening (Figure 1). She also had crusted post-chickenpox lesions on her body.

The ophthalmologic examination with a slit lamp showed no signs of herpetic keratitis or uveitis. An orbital CT scan was requested to rule out an associated orbital cellulitis, and it did not reveal any abscess. The diagnosis of ophthalmic shingles was made based on the appearance of the lesions and their segmental distribution along the nasal branch of the V1 dermatome.

**Figure 2:**
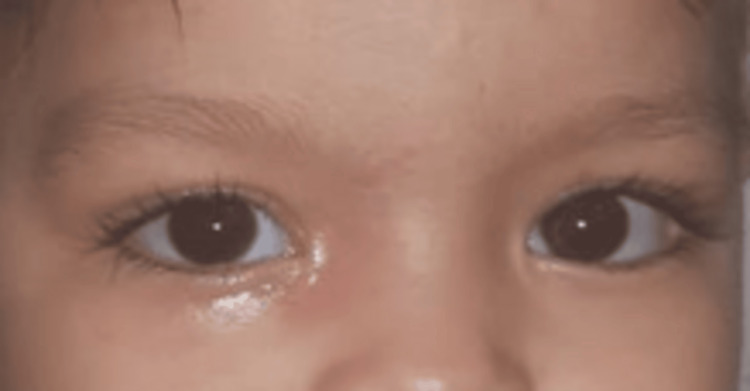
Disappearance of skin signs after treatment

The child was initially treated intravenously with acyclovir at a dose of 10 mg/kg every eight hours, along with local treatment. On day 3 of treatment, during a follow-up ophthalmologic examination, the presence of an inferior corneal ulcer (KPS) marked by fluorescein staining and a small inferior temporal ulceration (0.5 mm) was noted. At this stage, we added an antibiotic treatment consisting of ceftriaxone and metronidazole for 10 days, and gentamicin for three days. The clinical course showed improvement, with regression of the edema (Figure 2), and the child received acyclovir intravenously for two weeks, supplemented by one week of oral treatment.

A minimal immunological workup was conducted, including a complete blood count, human immunodeficiency virus serology, and quantitative measurements of immunoglobulins, all within normal limits except for lymphocytic-predominant leukocytosis (Table 1). The current follow-up period spans four years with no recurrence or postherpetic pain.

**Table 1:** Our patient's biological findings IgG: Immunoglobulin G, IgA: Immunoglobulin A, IgM: Immunoglobulin M, IgE: Immunoglobulin E, HIV: Human Immunodeficiency Virus

Laboratory parameter	Test results	Reference range
Hemoglobin (g/dl)	12.4	11-13.5
White blood cell (/µ)	18370	4000-10000
Lymphocyte (/µl)	10103	2000-4000
Neutrophil (/µl)	6180	1500-7000
Platelets (/µl)	400000	150000-400000
C-reactive protein (mg/l)	2.3	<5
IgG (g/l)	4.1	3.4-6.2
IgA (g/l)	1.1	0.33-1.2
IgM (g/l)	0.9	0.48-1.43
IgE (UI/ml)	25	<60
HIV1/2 (P24 antigen and HIV 1/2 antibodies screening)	0.21	<1

## Discussion

After respiratory transmission, the incubation period typically lasts for 14 days. Subsequently, the VZV spreads through the bloodstream to reach the skin and mucous membranes, which are the target organs, thereby triggering the primary infection (chickenpox) [1]. Following this primary infection, the VZV enters the sensory ganglia via hematogenous and/or neurogenic routes from the skin or mucous membranes, where it enters a latent state. Shingles is a localized reactivation that occurs when the virus exits this latent state, often due to changes in virus pathogenicity and/or cellular immunity. During this reactivation, the virus migrates along sensory nerve fibers until it reaches the skin [3].

Ophthalmic shingles is more common in the elderly than in children. It is characterized by its distinct clinical presentation and the high risk of ocular complications and severe pain. It represents between 10 and 30% of all cases of shingles. In children, this form is rare and generally associated with a more favorable prognosis, with few cases of postherpetic neuralgia after healing [1,4]. An Indian study revealed that out of 195 cases of shingles, 22 had ophthalmic shingles, of which 10% were in children [5].

Ocular involvement in children during the first four years of life is rare and potentially serious in terms of function if not treated early. In such cases, it is essential to investigate a history of chickenpox in the mother during pregnancy or an immunocompromised state [6]. In infants, shingles is most often the result of in utero transmission during chickenpox contracted by the mother during pregnancy. A few cases of infant shingles have been reported in infants with a history of maternal chickenpox during pregnancy [1]. In the literature, 26 cases of shingles in infants have been reported [7,8]. Among them, 15 appear to result from intrauterine exposure, while six cases had no history of previous exposure to the virus.

The term "ophthalmic shingles" is used when one of the three branches of the trigeminal nerve (V nerve) is affected: the ophthalmic, maxillary, and mandibular branches. Each branch innervates a specific area: the frontal branch covers half of the forehead and the inner part of the upper eyelid, the lacrimal branch concerns the temporomalar region and the outer part of the upper eyelid, while the nasal branch extends from the inner corner of the eye to the conjunctiva, the root of the nose, and the nasal septum [6].

The clinical diagnosis of ophthalmic shingles is generally straightforward. In the acute stage, its characteristics are well-defined. It manifests in 70% of cases with unilateral superficial pain in the trigeminal dermatome, presenting as burning and shooting pains. Additionally, a general infectious syndrome and palpable preauricular lymphadenopathy are often observed. 24 to 48 hours later, the rash appears: erythematous then papular then unilateral vesicular. Ocular manifestations may sometimes precede the skin rash and can compromise visual function, thus contributing to the severity of the condition. Biological diagnosis is usually unnecessary, but in case of uncertainty, a vesicular fluid sample can be taken to search for the presence of the VZV through immunofluorescence or culture. PCR is a rapid, specific, and highly sensitive method for detecting very low quantities of viral DNA in vesicular fluid and mononuclear cells from peripheral blood during the viremia period [6].

The ocular complications of ophthalmic shingles occur in 50 to 70% of cases, often with a guarded prognosis in children. They mainly include keratitis, conjunctivitis, uveitis, retinitis, retinal necrosis, glaucoma, and optic nerve necrosis. Although neurological complications are possible, they fortunately remain rare, including myelitis, meningoencephalitis, and motor and oculomotor paralysis, as well as bladder and digestive dysfunctions [9]. The severity of these complications necessitates ophthalmological management and follow-up to mitigate their consequences. A localized shingles outbreak may raise discussion concerning herpes simplex, particularly in the presence of recurrence at the same site. Additionally, in cases with few vesicles, eczema may also be considered in the differential diagnosis [1].

The treatment of ophthalmic shingles typically involves the use of oral or intravenous antivirals to reduce viral replication, along with anti-inflammatories and analgesics to relieve pain. Topical corticosteroids may be prescribed to reduce ocular inflammation, while moisturizing drops can help alleviate dry eye. However, it is important to note that corticosteroids should be used very carefully, especially during the chronic period, in conjunction with antibiotics and antiviral treatment, particularly when corneal stromal involvement and/or uveitis are present. Regular follow-up by an ophthalmologist is recommended to detect and treat any potential ocular complications. Early treatment is essential to reduce the risk of serious complications and improve outcomes [1,6].

## Conclusions

The particularity of our observation is the occurrence of ophthalmic shingles in a child under four years old, immunocompetent, who recently had chickenpox. Moreover, ophthalmic involvement remains a rare form in children but carries a risk of severe ocular complications in the absence of early antiviral treatment.
